# Hepatic arterial infusion chemotherapy with implantable arterial access port for advanced-stage hepatocellular carcinoma: a case report

**DOI:** 10.3389/fonc.2024.1401882

**Published:** 2024-05-16

**Authors:** Xin Jiang, Afaf Aljbri, Jiaxuan Liu, Liqi Shang, Yulong Tian, Haibo Shao

**Affiliations:** Department of Interventional Radiology, The First Affiliated Hospital of China Medical University, Shenyang, Liaoning, China

**Keywords:** hepatocellular carcinoma, FOLFOX-HAIC, anti-angiogenic therapy, immune checkpoint inhibitors, case report

## Abstract

**Background:**

Hepatocellular carcinoma (HCC) is a common gastrointestinal malignancy characterized by high incidence rates and a poor prognosis. Common treatment modalities include surgery, ablation, and transarterial chemoembolization (TACE). Hepatic arterial infusion chemotherapy (HAIC) has long been used in the treatment of unresectable liver cancer. In recent years, the combination of anti-angiogenesis therapy and immune checkpoint inhibitors has shown significant advances in the treatment of middle- and advanced-stage liver cancer. This report presents a case of HCC in which sustained benefits are achieved through a combination of HAIC of infusional oxaliplatin, leucovorin, and fluorouracil (FOLFOX), targeted therapy, and immunotherapy.

**Main body:**

A 64-year-old male patient was diagnosed with a parenchymal mass in the liver by a three-dimensional color ultrasound one month before admission, prompting consideration of liver cancer. Subsequently, computed tomography (CT) imaging performed at our hospital identified mass shadows in the right lobe of the liver and diffuse nodules throughout the liver, suggesting malignant lesions. Upon admission, the patient presented poor general health and baseline indicators. Following symptomatic treatment, the patient underwent a therapeutic regimen that combined transarterial infusion port FOLFOX-HAIC with Lenvatinib and Sintilimab. This combined treatment resulted in significant liver tumor necrosis and effectively managed the patient’s condition.

**Conclusion:**

The combined approach of using FOLFO-HAIC transarterial infusion alongside anti-angiogenesis therapy and immune checkpoint inhibitors has shown promising results that provide substantial benefits. This combined regimen has demonstrated the potential to improve treatment compliance among certain patients. Given these encouraging outcomes, further investigation into this combination therapy regimen is warranted to understand better its efficacy and potential broader applications in clinical settings.

## Introduction

According to the latest global cancer data released by the WHO in 2020, primary liver cancer ranks sixth in the incidence of malignant tumors and third in mortality rate (1). The onset of liver cancer often presents subtly, and while early-stage disease can be managed by resection, liver transplantation, or ablation, a considerable proportion of patients face incurable disease and poor prognosis (2). For patients at the Barcelona Clinic, liver cancer (BCLC), stage A-B with unresectable hepatocellular carcinoma (HCC), transarterial chemoembolization (TACE) is commonly chosen as the primary treatment; however, its efficacy depends heavily on tumor size. Treatment of large HCC of Child-Pugh class A-B (10 cm) remains challenging due to unsatisfactory outcomes (3). Hepatic arterial infusion chemotherapy (HAIC), which includes oxaliplatin, leucovorin, and fluorouracil (FOLFOX), targets middle to advanced HCC and offers substantial survival benefits (4). In Japan, HAIC has preferred for people with large HCC or portal vein tumor thrombosis (PVTT), particularly for severe cases of PVTT (5). In particular, the findings of a randomized phase 3 study comparing HAIC and TACE for extensive HCC indicate that HAIC contributes to improved survival outcomes for large HCCs (6). However, the survival benefit of FOLFOX-HAIC alone remained limited.

In recent years, anti-angiogenic therapy and immune checkpoint inhibitors have effectively treated advanced HCC. Phase 3 study in 2018 revealed that Lenvatinib, a representative agent in anti-angiogenic therapy compared to Sorafenib, exhibited non-inferior OS (13.6 vs. 12.3 months) and a higher objective response rate (ORR) of 18.8% (7). In recent years, immune checkpoint inhibitors have been the focus of research in treating advanced tumors and have also made significant progress in treating HCC. Sintilimab is an anti-programmed cell death protein (PD-1) monoclonal antibody with high anti-tumor activity in HCC (8). Clinical trials and reports have increasingly demonstrated the superior effectiveness of combination therapy over monotherapy. Therefore, we report that a patient with extensive unresectable HCC who received FOLFOX-HAIC with Lenvatinib and Sintilimab through an arterial infusion port achieved a sustained survival benefit.

## Case description

**Figure 1:**
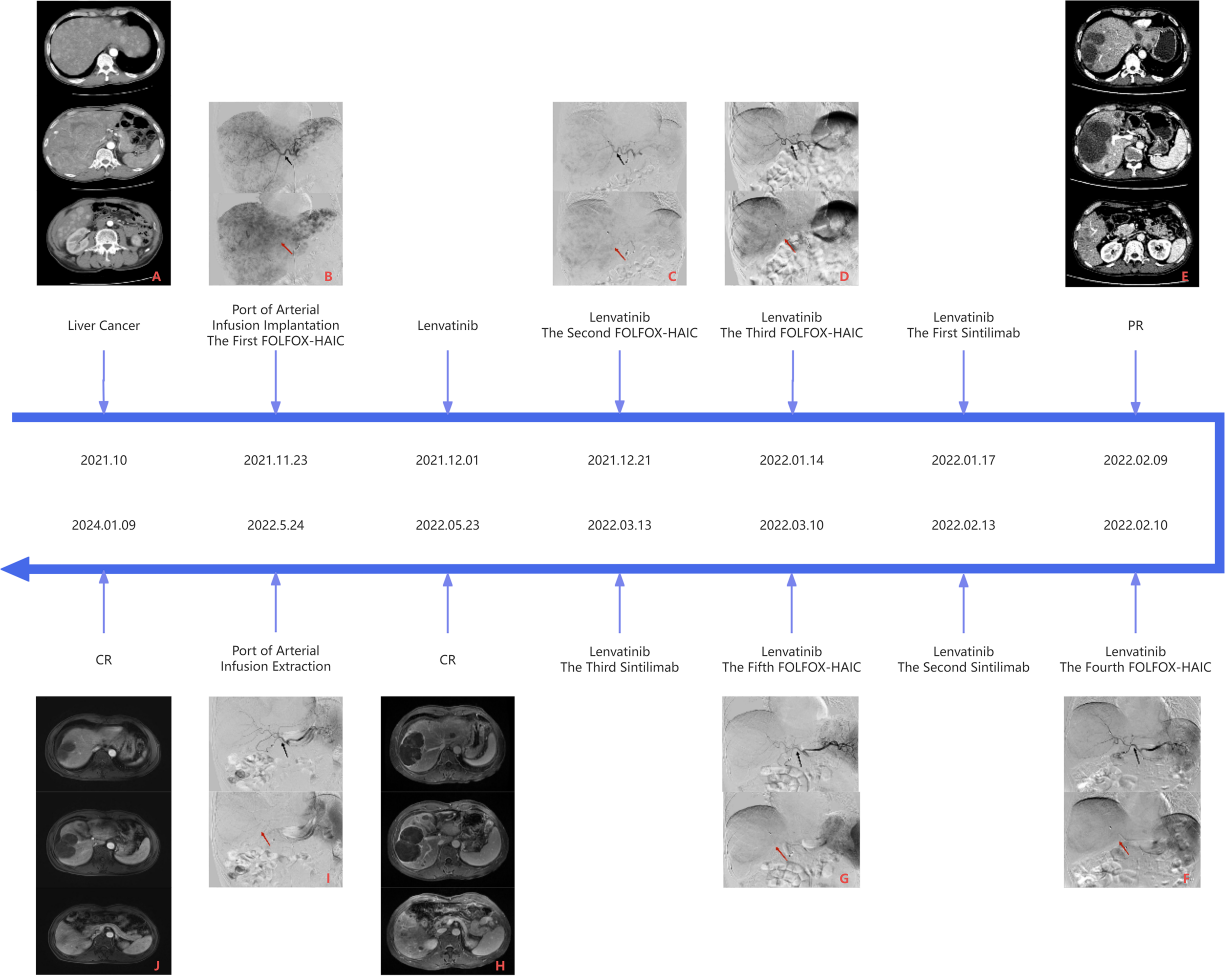
Panel **(A)** is the CT image of the liver before the first FOLFOX-HAIC, and panel B is the DSA image of the first FOLFOX-HAIC, both of which can see massive masses in the right lobe of the liver, multiple nodules in the liver, and apparent tumor staining. Panel **(E)** is the first efficacy assessment after three FOLFOX-HAIC treatments combined with Lenvatinib and Sintilimab. CT examination showed mild circular enhancement of giant tumors in the right lobe of the liver in the arterial stage, and some nodular lesions in the liver were mildly enhanced, but no new lesions were detected. Panel **(F)** is the DSA image of the fourth FOLFOX-HAIC, as shown in panel **(E)**. In contrast to panel **(B)**, the hepatic artery vessels (black arrow) become thinner, the volume of the tumor (red arrow) in the right lobe of the liver is significantly reduced, and the tumor staining is reduced considerably, which can reach partial response (PR) according to Response Evaluation Criteria in Solid Tumors (RECIST) v1.1. Panel **(H)** is the second evaluation of the efficacy after five FOLFOX-HAIC treatments combined with Lenvatinib and Sintilimab. A liver MRI examination showed fine line enhancement in the liver at the arterial stage, liquefaction necrosis in the original lesion area, and no new lesions. Panel **(I)** shows the angiography before removing the artery infusion port, where no noticeable tumor staining was observed. Compared with panel **(F)**, the blood vessels (black arrow) in the right lobe of the liver are further narrowed, and the volume of the masses (red arrow) in the right lobe of the liver is significantly slightly reduced, up to complete response (CR), and continued until the latest review.

The patient, a 64-year-old Chinese man, presented in November 2021 with a three-month history of diarrhea, fatigue, and lower extremity symptoms. On 10 November 2021, the patient underwent a CT (CT) examination at our hospital, revealing findings of a mass shadow in the right lobe of the liver and diffuse nodules throughout the liver, suggesting malignant liver tumors. In particular, the patient had a medical history of chronic hepatitis B virus (HBV) and is currently on antiviral therapy. The patient denied any history of underlying conditions such as hypertension, diabetes, or coronary heart disease; the patient also reported no family history of infection or cancer. Before admission, the patient had not received any treatment. The chronological progression of the entire case is illustrated in **Figure 1**.

## Diagnostic assessment

In November 2021, the patient underwent a CT examination that revealed multiple intrahepatic lesions. The maximum cross-sectional dimension of the giant mass shadow in the right liver lobe measured approximately 16.3 × 11.3 cm, accompanied by scattered nodules within the liver. These lesions demonstrated enhancement during the arterial phase of imaging, suggesting a stage B BCLC classification. The patient was then admitted for a routine examination (**Table 1**). The serum tumor marker test revealed a serum level of carbohydrate antigen 199 (CA199) of 70.90 U/ml, while alpha-fetoprotein (AFP) and carcinoembryonic antigen (CEA) fell within normal ranges. The indicators of liver function reflected poor liver function, and the Child-Pugh classification of liver function was B-grade. Consequently, preoperative and postoperative liver protection therapy was administered.

**Table 1:** The results of the blood test.

ITEM	The pre-treatment (2021.09)	The after treatment (2022.05)	NORMAL RANGE	UNIT
AFP	2.12	4.75	0.00-7.00	ng/mL
CEA	2.55	2.08	0.00-4.30	ng/mL
CA199	70.90	21.10	0.00-27.00	U/mL
AST	251	48	15-40	U/L
ALT	50	35	9-50	U/L
ALB	30.1	37.6	40.0-55.0	g/L
TBIL	40.6	13.4	0.0-26.0	umol/L
DBIL	29.6	3.9	0.0-8.0	umol/L
PT	12.9	13.5	11.0-13.7	s

**Figure 2:**
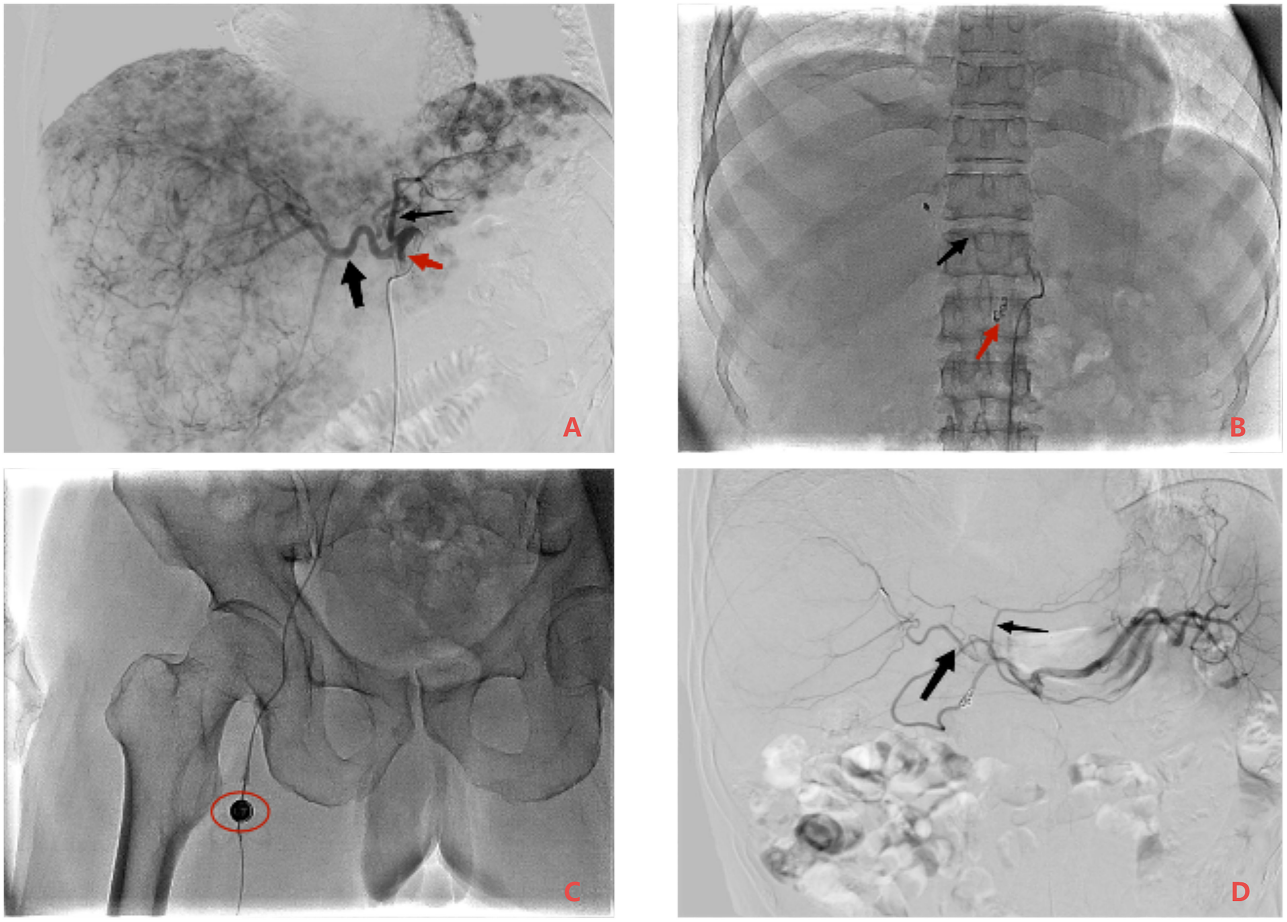
Comparison of DSA images before and after treatment and implantation of an arterial infusion port. Panel **(A)** is the image of the initial contrast. Large lesions in the right lobe of the liver and scattered lesions in the liver can be seen. Blood is supplied by the left hepatic artery (thin black arrow) and the right hepatic artery (thick black arrow), and the initial segment of the gastroduodenal artery (red arrow) can be seen. Panel **(B)** shows the position of the tip of the intrahepatic catheter at the port of arterial infusion (black arrow). In order to alleviate the adverse reactions after infusion, the gastroduodenal artery was selectively embolized with a spring ring (red arrow). Panel **(C)** shows the location and shape of the arterial infusion port (red circle). Panel **(D)** is the sixth contrast image. Compared with panel **(A)**, the left hepatic artery (thin black arrow) and the right hepatic artery (thick black arrow) were significantly thinner, and the tumor staining disappeared.

Based on the patient’s heavy tumor load and poor liver function, FOLFOX-HAIC therapy was selected once every 21 days through the infusion port of the femoral artery after a comprehensive assessment of the patient’s condition. On 23 November 2021, we performed hepatic arteriography under DSA guidance. The imaging revealed extensive lesions in the right liver lobe and multiple intrahepatic lesions supplied by both the left and right hepatic arteries, as shown in **Figure 2**.

To minimize postoperative adverse reactions from local chemotherapy drug infusions, we selectively embolized the gastroduodenal artery with spring coils. Subsequently, the arterial infusion port catheter was placed in the proper hepatic artery, with a lateral hole created 1 cm behind the tip to infuse the lesion in the left hepatic lobe. Finally, the artery infusion port was implanted subcutaneously 3 cm below the right groin, and liver arteriography was performed again with a 20G noninvasive needle through the artery infusion port, indicating that the catheter tip was not displaced and the catheter was unobtrusive. FOLFOX-HAIC was initiated through the femoral artery infusion port upon returning to the ward.

According to the body surface area of the patient, the specific regimen was calculated as oxaliplatin 150 mg (0–2h, 85mg/m2, 250ml/h), leucovorin 400mg (2–4h, 200mg/m2, 250ml/h), fluorouracil 3750 mg (subsequently 46–48h, 2500 mg/m2, 43 ml/h). During chemotherapy infusion, the patient had only mild liver pain related to oxaliplatin injection. On the second day after perfusion, the patient developed a fever, and relevant indicators were tested, indicating a high possibility of infection. After symptomatic treatment, the patient was discharged from the hospital and began to take 8 mg of Lenvatinib Mesylate capsule orally once a day.

In December 2021, the patient was readmitted to the hospital; the admission evaluation revealed that the patient’s liver function was still poor. Symptomatic liver protection treatment was maintained before surgery. Serum CA199 had decreased to normal levels, while AFP remained within the normal range, contradicting the indication of a high tumor load in the liver. Therefore, another serum tumor marker, prothrombin induced by vitamin K absence or antagonist II (PIVKA-II), was tested, revealing a>30000.00 mAU/mL result. On 21 December 2021, liver arteriography was performed in the interventional operating room using a 20G non-damaging needle through the infusion port of the femoral artery. Based on the DSA X-ray findings, the catheter appeared well positioned without discontinuity, and multiple tumor stains persisted in both the left and right lobes of the liver. However, compared to prior imaging, there was a reduction in tumor blood supply. Considering the patient’s myelosuppression (severe agranulocytosis) and decreased liver function after the previous treatment, the dose of oxaliplatin was halved for the second treatment with FOLFOX-HAIC. The infusion went smoothly without adverse reactions, and the patient was discharged the next day.

In January 2022, the patient was admitted to the hospital for the third time. The preoperative examination indicated that PIVKA-II decreased significantly to 637.50 mAU/ml. Other tests were all within the normal range. On 14 January 2022, liver arteriography demonstrated persistent tumor staining but with a further reduction in blood supply. The patient underwent a third FOLFOX-HAIC treatment following the same regimen as the previous session. The infusion proceeded smoothly without adverse reactions. Due to the substantial tumor burden, discussions with the patient’s family led to the decision to proceed with immunotherapy upon completion of this treatment. Before starting immunotherapy, the patient’s hypothyroidism indicators and myocardial enzyme profile were average. Post-intravenous infusion of Sintilimab injection 200 mg every 21 days, there were no apparent discomforts during the infusion, and the patient was discharged the following day.

After undergoing three cycles of FOLFOX-HAIC treatment, the patient underwent an enhanced CT examination on 9th February 2022 to assess the efficacy of the treatment. As seen from the DSA images in **Figures 1B–D**, hepatic artery vessels (black arrow) gradually became thinner and more apparent, intrahepatic lesion staining decreased significantly, and lesion volume (red arrow) decreased significantly. Partial tumor staining was still visible in **Figure 1D**. As shown in **Figure 1E**, the liver-enhanced CT scan showed mild circular enhancement of giant masses in the right lobe and mild enhancement of some nodules in the liver during the arterial stage. Compared with the images in September 2021, the lesion scope was reduced, the enhancement was decreased significantly, and no new lesions were found. Blood tests showed that PIVKA-II again significantly decreased and dropped to an average level of 36.19mAU/mL, AFP was still within the normal range, and liver function and coagulation function were normal. It is proved that FOLFOX-HAIC treatments combined with Lenvatinib and Sintilimab are effective, and this regimen can be continued. As shown in **Figures 1F, G**, hepatic arteriography sessions were conducted in February and March 2022, respectively, which revealed a successive decline in tumor staining and blood supply (red arrow), with blood vessels (black arrow) supplying the tumor, becoming thinner. The preoperative evaluation indicated good liver function, absence of myelosuppression, and normalized PIVKA-II levels. Subsequently, the patient underwent the fourth and fifth cycles of FOLFOX-HAIC treatment, following the same regimen as previously administered, and the infusion process proceeded smoothly without any adverse reactions. After FOLFOX-HAIC treatment, the patient underwent immunotherapy with the same regimen as before, experiencing no apparent discomfort during infusion.

**Figure 3:**
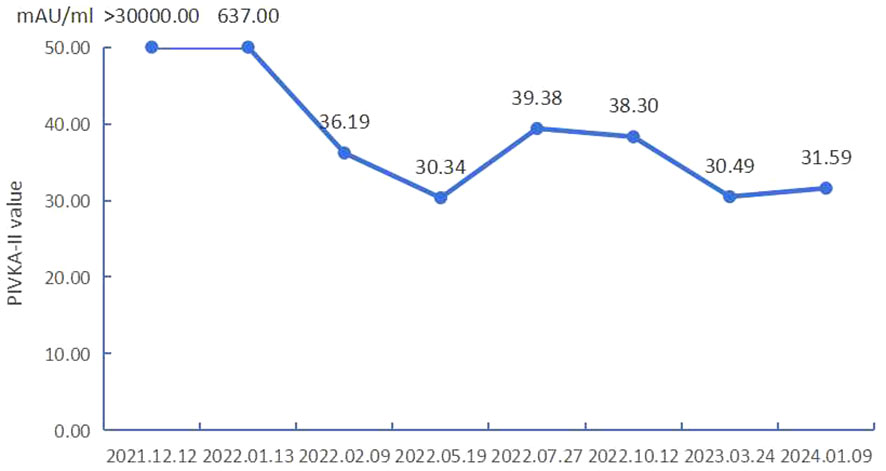
Line chart of serum PIVKA-II.

Blood tests in May 2022 indicated that PIVKA-II and AFP were in the normal range, as shown in **Table 1**; **Figure 3**, red blood cells and white blood cells were in the normal range, liver function was good, and coagulation function was normal. As shown in **Figure 1H**, Liver-enhanced magnetic resonance imaging (MRI) showed fine line enhancement within the lesion, demonstrating a reduced lesion area compared to the CT examination conducted in February 2022. On 24 May 2022, liver arteriography indicated the disappearance of tumor staining with no emergence of new lesions, as shown in **Figure 2**. The patient had an excellent physical and mental condition, a regular diet and sleep, and no adverse drug reactions. After the above comprehensive assessment, the medical team determined that the patient did not require a sixth FOLFOX-HAIC treatment. Consequently, the arterial infusion port and catheter were removed, and the wound was sutured under pressure and bandaged. After a day of observation without any discomfort, the patient was discharged.

After discharge, the patient took Lenvatinib 8 mg orally once daily and received Sintilimab 200 mg intravenously every 21 days. Enhanced liver MRI evaluations in July 2022, October 2022, March 2023, and January 2024 indicated stable lesions without significant changes. Serum tests revealed good liver function with no myelosuppressive reactions.

Throughout the treatment, there was a notable decrease in liver function and a myelosuppressive reaction after the initial two FOLFOX-HAIC treatments. However, these adverse effects were mitigated and improved after receiving symptomatic treatment. Subsequent treatments showed no apparent symptoms. The patient experienced a slight increase in blood pressure after initiating Lenvatinib. There were no adverse events related to immunotherapy.

## Discussion

In this report, we present the case of a patient diagnosed with unresectable HCC. The patient consented to FOLFOX-HAIC therapy, Lenvatinib targeting therapy, and Sintilimab immunotherapy after arterial infusion port implantation. Interestingly, the patient achieved a complete response after undergoing seven months of treatment, which persisted until this article’s submission. Progression-free survival has been calculated at 26 months.

According to the updated 2021 Japan Society of Hepatology (JSH) consensus statement and recommendations, TACE is the primary treatment for most asymptomatic patients exhibiting single or localized multifocal HCC, provided they possess well-preserved liver function. However, for large patients with HCC with multifocal biflobular nodules, fusion nodules, and Child-Pugh grade B, HAIC has become the preferred treatment, especially for patients with severe portal vein tumor thrombosis (PVTT) (5, 9). Since the liver artery primarily supplies HCC, HAIC is the direct and continuous administration of chemotherapy drugs to the tumor via a percutaneous arterial catheter, increasing the local drug concentration of cancer, avoiding the first-pass effect, and minimizing systemic adverse reactions of drugs. Additionally, a few chemotherapy drugs subsequently enter the systemic circulation and have systemic antitumor effects. As a result, HAIC can be considered a systemic treatment method with enhanced local efficacy (10).

Moreover, He et al.’s prospective, nonrandomized study illustrated that FOLFOX-HAIC exhibits a higher Objective Response Rate (ORR) than TACE when treating large liver cancer. Specifically, HAIC based on oxaliplatin could demonstrate superior effectiveness to cisplatin-based HAIC for HCC (11). Due to single intubation, patients undergoing HAIC must remain in a supine position for more than 48 hours and cannot be turned at will. Based on the patient’s history of poor compliance and treatment experience, implantable systems have been widely used in tumor perfusion therapy over the past few decades (12). We opt for FOLFOX-HAIC through a femoral artery infusion port with the patient’s and their family’s consent. In this case, there are two clever designs throughout the operation. One is that we created a lateral hole 1 cm behind the catheter tip, which could simultaneously inject multiple lesions of both lobes and increase the stability of the catheter. The other is implanted in the arterial infusion port in a minimally invasive way, with minor trauma and high strength. Implementing the infusion port into the artery avoids multiple punctures and catheterizations in the femoral artery, significantly reducing the time to subsequent surgery. Importantly, this method does not restrict patient activities during treatment, improving compliance with numerous long-term HAIC regimens.

In recent years, combining immune checkpoint inhibitors and anti-angiogenic agents has markedly improved the treatment of unresectable HCC. Lenvatinib was approved in 2018 as a first-line treatment for advanced HCC (7). In particular, the immune checkpoint inhibitor Sintilimab has demonstrated potent antitumor activity in various cancers (8). Anti-VEGF therapy is crucial in reducing VEGF-induced immunosuppression within tumors and their microenvironment. It can potentially increase the effectiveness of anti-PD-1 and antiprogrammed death ligand 1 (PD-L1) treatments by counteracting VEGF-mediated immunosuppression and encouraging T-cell infiltration into tumors (13). The IMbrave150 trial established the superiority of combination therapies Atezolizumab and Bevacizumab over sorafenib monotherapy. This combination exhibited a markedly high objective response rate (ORR) of 30% and unprecedented overall survival (OS) of 19.2 months compared to 13.4 months with sorafenib monotherapy (p<0.001) (14). Furthermore, a phase 2 clinical study highlighted the efficacy of Sintilimab in combination with Lenvatinib. It reported an ORR of 36.1% in patients with medium-advanced or locally advanced HCC, showing a higher response rate than previously reported results from single-agent anti-PD-1 or Lenvatinib therapy. This underscores the potent antitumor activity achieved by combining Sintilimab and Lenvatinib (15).

Antiangiogenic drugs are expected to enhance the efficacy of HAIC, primarily by improving tumor vascular permeability and reducing tumor interstitial pressure. This improvement in the tumor microenvironment benefits from better distributing the chemotherapy drugs (5). A randomized Phase 3 trial was conducted among patients with advanced HCC complicated by portal vein tumor thrombosis that compared Sorafenib alone versus Sorafenib combined with FOLFOX-HAIC. The results showed that the combination therapy significantly extended overall survival (OS) (7.13 vs. 13.37 months, p<0.001) and exhibited a considerably higher response rate compared to the monotherapy group (51% vs. 3%, p<0.001) (4). Immunosuppressants demonstrate synergistic effects with HAIC, potentially inducing substantial local immune modulation within the HCC microenvironment (16). Furthermore, Lenvatinib and PD-1 inhibitors could improve chemotherapy drug delivery by promoting vascular normalization (17). In the study comparing the combined treatment of Lenvatinib, Toripalimab, and HAIC with Lenvatinib monotherapy, patients subjected to the combination therapy exhibited substantial improvements in various parameters. In particular, the combination therapy group achieved progression-free survival (PFS) of 11.1 months compared to 5.1 months for monotherapy (p<0.001). Similarly, overall survival (OS) improved markedly in the combination treatment group compared to monotherapy (not achieved vs 11 months, p<0.001). Furthermore, the combination therapy group showed higher objective response rates (ORR) according to the RECIST criteria (59.2% vs. 9.3%, p<0.001) and the m-RECIST criteria (67.6% vs. 16.3%, p<0.001). The high ORR and PFS observed in the combination treatment group might be due to synergistic antitumor effects (18).

The patient was treated with the combination of Lenvatinib after the completion of the first FOLFOX-HAIC and the combination of Sintilimab and Lenvatinib after the completion of the third FOLFOX-HAIC. According to RESIST1.1, PD was achieved after three FOLFOX-HAICs, CR was achieved after five FOLFOX-HAICs, and the arterial infusion port and catheter were easily removed. No AE related to the infusion port occurred during treatment. After combination therapy, the tumor supply vessels gradually narrowed, and the blood supply gradually decreased. All liver lesions showed necrosis, the maximum tumor volume reduced significantly, other lesions lost activity, and liver function slowly recovered. While on oral Lenvatinib, the patient experienced elevated blood pressure, which was managed with antihypertensive medication.

Moreover, there were no adverse reactions associated with immunotherapy. At the same time, throughout treatment, we also found that the patient’s AFP was consistently within the normal range, but the PIVKA-II levels were extremely high. Some researchers suggest that PIVKA-II may be more beneficial than AFP in HBV-associated HCC. Increased levels of PIVKA-II were associated with larger and more aggressive tumors, intrahepatic metastases, and recurrence after treatment and were significantly correlated with tumor size (P < 0.01) (19, 20). In this case, the content of PIVKA-II was extremely high for the first time and decreased significantly during treatment (**Figure 3**). In the corresponding image data, tumor enhancement decreased significantly.

In summary, the patient completed five FOLFOX-HAIC therapies at the arterial infusion port, during which the treatment combined with Lenvatinib and Sintilimab, as well as antiviral and symptomatic treatment, significantly decreased, tumor activity disappeared, and liver function recovered, achieving a perfect therapeutic effect. However, it is still being determined which specific treatment plays a key role and whether it can achieve the same impact on other patients deserves further research. Moreover, combination therapy is expensive, the drug side effects are large, and the treatment effect varies from person to person, so more clinical studies are needed to prove the effectiveness of this treatment.

## Data availability statement

The original contributions presented in the study are included in the article/supplementary material. Further inquiries can be directed to the corresponding authors.

## Ethics statement

The studies involving humans were approved by The First Affiliated Hospital of China Medical University. The studies were conducted in accordance with the local legislation and institutional requirements. This article is a retrospective case report and no informed consent is required. Written informed consent was obtained from the individual(s) for the publication of any potentially identifiable images or data included in this article.

## Author contributions

XJ: Conceptualization, Data curation, Investigation, Writing – original draft. HS: Data curation, Funding acquisition, Methodology, Resources, Supervision, Visualization, Writing – review & editing. YT: Data curation, Methodology, Supervision, Writing – review & editing. AA: Writing – original draft. JL: Methodology, Software, Writing – original draft. LS: Formal analysis, Software, Writing – original draft.

## References

[1] Sung H Ferlay J Siegel RL Laversanne M Soerjomataram I Jemal A . Global cancer statistics 2020: GLOBOCAN estimates of incidence and mortality worldwide for 36 cancers in 185 countries. CA Cancer J Clin. (2021) 71:209–49. doi: 10.3322/caac.21660 33538338

[2] Brown ZJ Tsilimigras DI Ruff SM Mohseni A Kamel IR Cloyd JM . Management of hepatocellular carcinoma: A review. JAMA Surg. (2023) 158:410–20. doi: 10.1001/jamasurg.2022.7989 36790767

[3] Li QJ He MK Chen HW Fang WQ Zhou YM Xu L . Hepatic arterial infusion of oxaliplatin, fluorouracil, and leucovorin versus transarterial chemoembolization for large hepatocellular carcinoma: A randomized phase III trial. J Clin Oncol. (2022) 40:150–60. doi: 10.1200/jco.21.00608 34648352

[4] He M Li Q Zou R Shen J Fang W Tan G . Sorafenib plus hepatic arterial infusion of oxaliplatin, fluorouracil, and leucovorin vs sorafenib alone for hepatocellular carcinoma with portal vein invasion: A randomized clinical trial. JAMA Oncol. (2019) 5:953–60. doi: 10.1001/jamaoncol.2019.0250 PMC6512278 31070690

[5] Kudo M Kawamura Y Hasegawa K Tateishi R Kariyama K Shiina S . Management of hepatocellular carcinoma in Japan: JSH consensus statements and recommendations 2021 update. Liver Cancer. (2021) 10:181–223. doi: 10.1159/000514174 34239808 PMC8237791

[6] Tsai WL Sun WC Chen WC Chiang CL Lin HS Liang HL . Hepatic arterial infusion chemotherapy vs transcatheter arterial embolization for patients with huge unresectable hepatocellular carcinoma. Med (Baltimore). (2020) 99:e21489. doi: 10.1097/md.0000000000021489 PMC7593048 32769883

[7] Kudo M Finn RS Qin S Han KH Ikeda K Piscaglia F . Lenvatinib versus sorafenib in first-line treatment of patients with unresectable hepatocellular carcinoma: a randomised phase 3 non-inferiority trial. Lancet. (2018) 391:1163–73. doi: 10.1016/s0140-6736(18)30207-1 29433850

[8] Ren Z Xu J Bai Y Xu A Cang S Du C . Sintilimab plus a bevacizumab biosimilar (IBI305) versus sorafenib in unresectable hepatocellular carcinoma (ORIENT-32): a randomised, open-label, phase 2–3 study. Lancet Oncol. (2021) 22:977–90. doi: 10.1016/s1470-2045(21)00252-7 34143971

[9] Lyu N Wang X Li JB Lai JF Chen QF Li SL . Arterial chemotherapy of oxaliplatin plus fluorouracil versus sorafenib in advanced hepatocellular carcinoma: A biomolecular exploratory, randomized, phase III trial (FOHAIC-1). J Clin Oncol. (2022) 40:468–80. doi: 10.1200/jco.21.01963 34905388

[10] Obi S Sato S Kawai T . Current status of hepatic arterial infusion chemotherapy. Liver Cancer. (2015) 4:188–99. doi: 10.1159/000367746 PMC4608627 26674592

[11] He MK Le Y Li QJ Yu ZS Li SH Wei W . Hepatic artery infusion chemotherapy using mFOLFOX versus transarterial chemoembolization for massive unresectable hepatocellular carcinoma: a prospective non-randomized study. Chin J Cancer. (2017) 36:83. doi: 10.1186/s40880-017-0251-2 29061175 PMC5654007

[12] Thiels CA D'Angelica MI . Hepatic artery infusion pumps. J Surg Oncol. (2020) 122:70–7. doi: 10.1002/jso.25913 PMC9014308 32215927

[13] Voron T Colussi O Marcheteau E Pernot S Nizard M Pointet AL . VEGF-A modulates expression of inhibitory checkpoints on CD8+ T cells in tumors. J Exp Med. (2015) 212:139–48. doi: 10.1084/jem.20140559 PMC4322048 25601652

[14] Cheng AL Qin S Ikeda M Galle PR Ducreux M Kim TY . Updated efficacy and safety data from IMbrave150: Atezolizumab plus bevacizumab vs. sorafenib for unresectable hepatocellular carcinoma. J Hepatol. (2022) 76:862–73. doi: 10.1016/j.jhep.2021.11.030 34902530

[15] Xu J Shen J Gu S Zhang Y Wu L Wu J . Camrelizumab in combination with apatinib in patients with advanced hepatocellular carcinoma (RESCUE): A nonrandomized, open-label, phase II trial. Clin Cancer Res. (2021) 27:1003–11. doi: 10.1158/1078-0432.Ccr-20-2571 33087333

[16] Fu Y Peng W Zhang W Yang Z Hu Z Pang Y . Induction therapy with hepatic arterial infusion chemotherapy enhances the efficacy of lenvatinib and pd1 inhibitors in treating hepatocellular carcinoma patients with portal vein tumor thrombosis. J Gastroenterol. (2023) 58:413–24. doi: 10.1007/s00535-023-01976-x 36894804

[17] Shigeta K Datta M Hato T Kitahara S Chen IX Matsui A . Dual programmed death receptor-1 and vascular endothelial growth factor receptor-2 blockade promotes vascular normalization and enhances antitumor immune responses in hepatocellular carcinoma. Hepatology. (2020) 71:1247–61. doi: 10.1002/hep.30889 PMC7000304 31378984

[18] He MK Liang RB Zhao Y Xu YJ Chen HW Zhou YM . Lenvatinib, toripalimab, plus hepatic arterial infusion chemotherapy versus lenvatinib alone for advanced hepatocellular carcinoma. Ther Adv Med Oncol. (2021) 13:17588359211002720. doi: 10.1177/17588359211002720 33854567 PMC8010824

[19] Seo SI Kim HS Kim WJ Shin WG Kim DJ Kim KH . Diagnostic value of PIVKA-II and alpha-fetoprotein in hepatitis B virus-associated hepatocellular carcinoma. World J Gastroenterol. (2015) 21:3928–35. doi: 10.3748/wjg.v21.i13.3928 PMC4385540 25852278

[20] Feng H Li B Li Z Wei Q Ren L . PIVKA-II serves as a potential biomarker that complements AFP for the diagnosis of hepatocellular carcinoma. BMC Cancer. (2021) 21:401. doi: 10.1186/s12885-021-08138-3 33849479 PMC8045263

